# A Case of Systemic Lupus Erythematosus and Antineutrophil Cytoplasmic Antibodies-Associated Vasculitis Overlap Syndrome

**DOI:** 10.1155/2021/6690658

**Published:** 2021-01-07

**Authors:** Hiroyuki Hounoki, Koichiro Shinoda, Atsushi Matsui, Maiko Okumura, Satoshi Yamaguchi, Kota Kakeshita, Hidenori Yamazaki, Tsutomu Koike, Teruhiko Makino, Kazuyuki Tobe

**Affiliations:** ^1^First Department of Internal Medicine, University of Toyama, 2630 Sugitani, Toyama 930-0194, Japan; ^2^Second Department of Internal Medicine, University of Toyama, 2630 Sugitani, Toyama 930-0194, Japan; ^3^Department of Dermatology, Graduate School of Medicine and Pharmaceutical Sciences, University of Toyama, 2630 Sugitani, Toyama 930-0194, Japan

## Abstract

An overlap of systemic lupus erythematosus (SLE) and antineutrophil cytoplasmic antibodies- (ANCA-) associated vasculitis (AAV) is extremely rare: approximately 40 cases have been reported to date. A literature review indicates that they are more common in women in their forties, and simultaneous onset has been reported in 69% of cases. In addition, both lupus nephritis and ANCA-associated glomerulonephritis were observed on renal biopsy. This report presents the case of a 35-year-old woman with an 8-month history of polyarthralgia who was admitted to our hospital. She was diagnosed with SLE due to typical clinical presentation of the disease: polyarthritis, lymphocytopenia, hypocomplementemia, presence of antinuclear and anti-dsDNA antibodies, and proteinuria. However, purpura were scattered, and the titer of antimyeloperoxidase-antineutrophil cytoplasmic antibodies (MPO-ANCA) was high. A skin biopsy revealed leukocytoclastic vasculitis that involved poor immune complex deposition. A renal biopsy showed necrotizing glomerulonephritis with cellular and fibrocellular crescent formation that involved deposition of IgM and C3c only in the mesangial area and the peripheral capillaries. Additionally, no electron-dense deposits were observed under electron microscopy. These pathological findings were consistent with AAV rather than with SLE. Therefore, we finally diagnosed the patient with both SLE and microscopic polyangiitis. After treatment with methylprednisolone and intravenous cyclophosphamide pulse therapies, renal function improved and MPO-ANCA levels decreased. In cases of suspected overlap between SLE and AAV, appropriate diagnosis and treatment are important.

## 1. Introduction

Although systemic lupus erythematosus (SLE) and antineutrophil cytoplasmic antibodies- (ANCA-) associated vasculitis (AAV) share a common organ involvement, such as the joints, skin, and kidney, they are different diseases with distinctive clinical findings in terms of age, sex, disease-specific autoantibodies, and pathology. Moreover, it has long been reported that antimyeloperoxidase-antineutrophil cytoplasmic antibodies (MPO-ANCA) are detected in patients with SLE [1]. According to a review by Sen and Isenberg, the positive rate of p-ANCA is 9.5 to 31.4% and the positive rate of MPO-ANCA is 0 to 23.8%, although no characteristic clinical features in cases with MPO-ANCA have been described [2]. However, recent reports have discussed the pathological role of MPO-ANCA in lupus nephritis (LN). Patients positive for MPO-ANCA are characterized by a high level of activity on renal histological examination and poor renal function [3, 4]. Nonetheless, the occurrence of these two conditions in a single patient is extremely rare, with only 40 cases reported in the literature. Hervier et al. proposed SLE and AAV as an overlapping syndrome [5, 6]. We report, herein, a case of SLE/AAV overlap diagnosed through serological and pathological examinations in a 35-year-old woman with polyarthralgia, skin rash, and renal impairment.

## 2. Case Presentation

**Figure 1:**
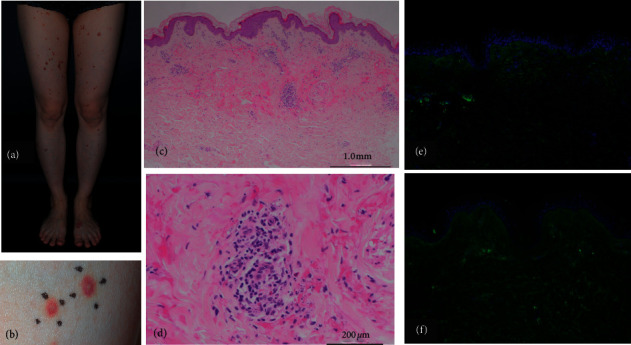
Skin lesions in this case. Purpura scattering on the legs (a) and magnified image of the purpura (b). Examination of punch biopsy specimen from the left lower leg (c, d) showing infiltration of inflammatory cells including neutrophils around small vessels in the dermis, which is consistent with leukocytoclastic vasculitis (hematoxylin-eosin stain). Immunofluorescence showing poor immune complex deposition ((e) IgM; (f) C3c).

**Figure 2:**
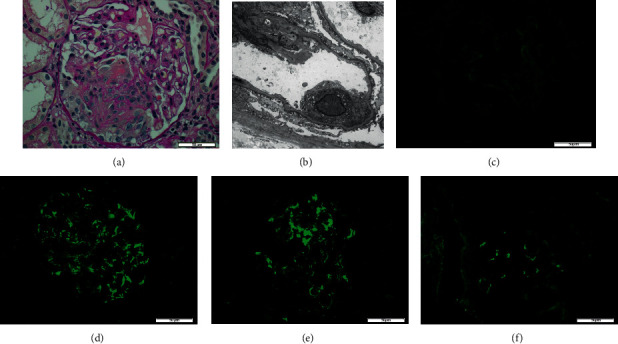
Renal biopsy findings. Eleven of 21 glomeruli presented crescent formation including five cellular crescents and six fibrocellular crescents in light microscope. (a) Renal biopsy revealing necrotizing glomerulonephritis with cellular and fibrocellular crescent formations. No wire loop lesion or nuclear fragmentation is present (hematoxylin-eosin stain). Immunofluorescence staining showing only deposition of IgM and C3c in mesangial areas and peripheral capillaries: (c) IgG, (d) IgM, (e) C3c, and (f) C1q. Electron microscopy showing no electron-dense deposits (b).

**Figure 3:**
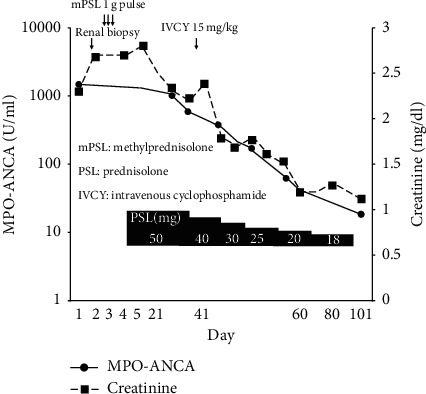
Clinical course of this case. The combination therapy with mPSL and IVCY was effective for improving the renal function of the patient and decreasing the titer of MPO-ANCA in this case. MPO-ANCA is displayed logarithmically. mPSL = methylprednisolone, PSL = prednisolone, and IVCY = intravenous cyclophosphamide.

A 35-year-old Japanese woman was admitted to our hospital for purpura and acute renal failure. She had an 8-month history of morning stiffness, and polyarthralgia in the bilateral shoulders, elbows, and hands. The patient reported purpura 4 days before admission. On physical examination, she was afebrile and her blood pressure was 106/67 mmHg. In addition, purpura lesions were scattered on the upper extremities, buttocks, and legs (Figures 1(a) and 1(b)). The patella of the left knee joint was floating because of articular fluid. A laboratory test results revealed (Table 1) white blood count, 3100/*μ*L; lymphocyte count, 1080/*μ*L; hemoglobin level, 8.5 mg/dl; albumin level, 3.2 mg/dl; urea nitrogen level, 31 mg/dl; creatinine, 2.3 mg/dl; C-reactive protein level, 0.35 mg/dl; and increased erythrocyte sedimentation rate, 59 mm/h. In addition, urine analysis showed proteinuria (1.7 g/gCr) and active urine sediment containing dysmorphic red blood cells. Serum complement levels were low, antinuclear antibodies (ANA) were positive, and anti-double-stranded DNA antibodies (anti-ds-DNA antibodies) were slightly elevated on screening by the enzyme-linked immunosorbent assay (ELISA) and radioimmunoassay method. Moreover, anti-SS-A, anti-SS-B antibody, and MPO-ANCA levels were elevated. The patient did not complain of either xerostomia or xerophthalmia, and salivary secretion was normal at 4.5 g/2 min measured by the Saxon test. Tear secretion (20 mm/min in the right eye, 18 mm/min in the left eye) measured by the Schirmer test and ocular surface staining with the fluorescein test was also normal. Labial salivary gland biopsies could not be performed without the patient's consent. Therefore, a diagnosis of Sjogren's syndrome (SS) could not be made. The patient was initially diagnosed with SLE due to typical clinical features: polyarthritis, lymphocytopenia, presence of antinuclear and anti-dsDNA antibodies, and proteinuria. However, MPO-ANCA levels were significantly high, and the skin manifestation was not typical of SLE. A skin biopsy revealed leukocytoclastic vasculitis involving poor immune complex deposition, which is typical of AAV rather than SLE (Figures 1(c)–1(f)). A renal biopsy revealed necrotizing glomerulonephritis with cellular and fibrocellular crescent formations. No wire-loop lesions or nuclear fragmentation that are detected in LN were present (Figure 2(a)). Immunofluorescence staining showed only deposition of immunoglobulin M (IgM) and C3c in the mesangial areas and peripheral capillaries (Figure 2(c)–2(f)). Electron microscopy showed no electron-dense deposits (Figure 2(b)). Renal histopathological findings resembled ANCA-associated glomerulonephritis rather than LN. The diagnosis of microscopic polyangiitis (MPA) was made based on these biopsy findings and MPO-ANCA positivity. Therefore, we finally diagnosed the patient with overlapping of SLE and MPA. Methylprednisolone pulse therapy was administered followed by oral prednisolone (PSL) (50 mg/day) and cyclophosphamide (15 mg/kg) intravenously. After treatment, arthralgia, renal function, proteinuria, and skin manifestations improved (Figure 3). Five years from the diagnosis, the patient is in near remission except for occasional hypocomplementemia treated with PSL 3 mg/day, mizoribine 150 mg/day, and tacrolimus 2 mg/day.

## 3. Discussion

This case involved polyarthritis, leukopenia, lymphopenia, the presence of antinuclear and anti-ds-DNA antibodies, and proteinuria. Our patient was initially diagnosed with SLE considering her sex and age. However, MPO-ANCA was markedly elevated and based on the results of the renal and skin biopsies, and the lesions were suggestive of AAV rather than SLE. We needed to consider whether this case could be diagnosed as a young-onset MPA with positive antinuclear and anti-ds-DNA antibodies or as an overlap between MPA and SLE. In particular, SLE and MPA have common clinical symptoms, such as arthralgia, skin eruption, nephritis, and fever. In this case, the final diagnosis depended on which disease is considered to be responsible for the joint, skin, and renal lesions. Based on the typical pathological findings of AAV from the skin and renal lesions and markedly elevated MPO-ANCA levels, the diagnosis of MPA could be made according to the Chapel Hill 2012 AAV definition criteria. On the other hand, based on an 8-month history of polyarthralgia, leukopenia, lymphopenia, positive ANA, positive anti-ds-DNA, and low complement (C3 and CH50), the patient was diagnosed with SLE according to the 1997 revised American College of Rheumatology (ACR) classification criteria (she had 4 items: polyarthritis, leukopenia/lymphopenia, positive antinuclear antibodies, and positive anti-ds-DNA antibodies); the Systemic Lupus International Collaborating Clinics classification criteria (she had 5 items: polyarthritis with morning stiffness, leukopenia, positive antinuclear antibodies, positive anti-ds-DNA antibodies, and low complement); and the 2016 revised European League Against Rheumatism/ACR classification criteria (total score 18: positive antinuclear antibodies at a titer of ≥1 : 80, polyarthritis with morning stiffness, leukopenia, low C3, and positive anti-ds-DNA antibodies). Arthralgia is a symptom of both SLE and MPA; thus, it was difficult to determine whether arthritis noted in this case was due to SLE or MPA. Arthritis is one of the most common symptoms observed in SLE and frequently present at the time of diagnosis and can precede the diagnosis of SLE by months or years [7]. Finally, as the patient had an 8-month history of SLE that was later complicated with AAV involving skin and renal vasculitis, we comprehensively identified this case as SLE/AAV (MPA) overlapping syndrome.

Hervier et al. reported that SLE associated with AAV may be a group of diseases called SLE/AAV overlapping syndrome, rather than an incidental concurrence of the two diseases [5]. Jarrot et al. examined the cases in which glomerulonephritis was found through renal biopsy and analyzed overlapping autoantibodies and SLE/AAV overlapping syndrome cases [6]. In the analysis of SLE/AAV overlapping syndrome, a total of 39 cases were accumulated. Ninety percent of the SLE/AAV overlapping syndrome cases involved women, with an average age of onset of 40 years for SLE and 42 years for AAV, indicating a much younger age than that of AAV. Simultaneous onset of the two diseases was found in 69% of cases, and rapidly progressive glomerulonephritis was found in 92% of cases. In the autoantibody test, 95% of the cases were positive for p-ANCA and 82% were positive for MPO-ANCA using ELISA. These clinical features are consistent with the findings in our case. Pathological examination of renal tissue was performed in eight patients examined at their respective institutions, three of whom were diagnosed with LN and five with AAV. However, in some patients pathologically diagnosed with LN, only a slight amount of immune complex deposition was observed, whereas in some patients diagnosed with AAV, a moderate amount of immune complex deposition was noted. Jarrot et al. mentioned that the absence of electron microscopy of kidney biopsies prevented a detailed analysis of the localization of immune complex deposits in their study [6]. However, several cases have been reported in which it was difficult to distinguish between the two diseases using an electron microscope, and there is a possibility that the renal lesions may be overlapping [8–11]. In our case, the pathology of crescentic glomerulonephritis was not a full-house pattern, although it could not be interpreted as a complete pauci-immune pattern because of the positive findings of C3c and IgM staining in indirect fluorescent staining. However, electron microscopy revealed that the immune complexes, which were electron-dense deposits, were scarce, and the renal lesions in this case were mainly because of AAV rather than LN.

To understand how the SLE/AAV overlapping syndrome is different from the situation of the presence of overlapping autoantibodies, the clinical characteristics with and without ANCA in 40 cases of LN and with and without ANA in 61 cases of pauci-immune glomerulonephritis (GN) were compared [6]. The presence of ANCA in LN did not differ in either age or renal pathological findings, and the presence of ANA in AAV also did not differ in either age or renal pathological findings. Overall, among the 101 patients from this cohort, 57 (56.5%) had typical LN or typical pauci-immune GN without overlapping antibodies; 12 (12%) had typical LN and were ANCA-positive; 30 (29.5%) had typical pauci-immune GN and were ANA-positive; and only 2 (2%) fulfilled the classification criteria for both SLE and AAV. These findings suggest that overlapping autoantibodies is different from true overlapping syndrome.

Pathological investigation was required to determine whether the skin lesions in this case were due to SLE or AAV. Our patient presented with purpura, and pathological findings revealed leukocytoclastic vasculitis. These findings were consistent with the characteristics of AAV. On the other hand, in SLE, concomitant cutaneous vasculitis, predominantly small vessel vasculitis has been reported in 19–28% of patients, and the main skin manifestations include palpable purpura, petechiae, livedo reticularis, and so on [12]. Finally, we concluded that this skin manifestation was due to AAV because of scarce immunoglobulin deposition around the small vessels and a negative lupus band test on indirect fluorescent antibody testing.

Several hypotheses on the mechanisms of the development of two or more diseases in a single patient were suggested. One of these is polyautoimmunity, a concept that refers to the development of multiple autoimmune diseases in a single patient. Rojas-Villarraga et al. reported that 40.6% of patients with SLE have polyautoimmunity. Autoimmune diseases associated with SLE include autoimmune thyroid disease, SS, and antiphospholipid antibody syndrome; these four diseases form a representative cluster of autoimmunity. However, overlap between vasculitis and this cluster is rare [13]. Our case had anti-SS-A and SS-B antibodies without sicca symptoms and antithyroid peroxidase and thyroglobulin antibodies with subclinical hypothyroidism, suggesting a common genetic background for this cluster. Martín-Nares et al. reported that, in 28 (11.3%) of 247 patients, AAV was associated with other autoimmune diseases, with rheumatoid arthritis being the most common (39%), followed by SS and scleroderma in 14% and SLE in only 2 patients [14].

Neutrophil activation is another possible mechanism for the overlap between SLE and AAV. Although MPO-ANCA has been shown to play a major role in the pathogenesis of AAV [15], neutrophil extracellular traps (NETs) and concomitant NETosis are important for the production of MPO-ANCA [16]. NETs are an immune response in biological defense, although their clearance is important, with DNase1 playing a critical role in their degradation [17]. Reduced serum DNase1 activity is observed in both AAV and SLE [18, 19]. Propylthiouracil, an antithyroid agent which is the causative agent of drug-induced lupus and vasculitis, has been reported to produce abnormal conformation and impaired degradation of NETs by DNase1, supporting the development of SLE/AAV overlapping syndrome via NETs [20]. Prior infection and drug exposure have not been proven in our case, although we are hypothesizing on the involvement of NETs as a mechanism for the simultaneous onset of the two diseases.

In conclusion, if vasculitis is suspected in patients with SLE, ANCA measurement and appropriate tissue biopsies are recommended. Treatment strategy should be optimized based on pathological and immunological findings to improve the prognosis of SLE/AAV overlapping syndrome.

## Figures and Tables

**Table 1:** Laboratory results of this case.

Urinalysis			Biochemistry/immunology					
Urine protein		(2+)	TP	7.5	g/dL	FANA	1 : 320 (speckled)	
Occult blood		(3+)	Alb	3.2	g/dL	Anti-dsDNA Ab (ELISA)	18.2	U/mL
Urine WBC		10–19/HF	BUN	31	mg/dL	Anti-dsDNA Ab (RIA)	9	U/mL
Urine RBC (with dysmorphic RBC)		>100/HF	Creatinine	2.3	mg/dL	Anti-Sm Ab	0.5	U/mL
Casts (red blood cell, granular, waxy)		(+)	eGFR	20.8	mL/min/1.73 m^2^	Anti-RNP Ab	<7.0	U/mL
U-TP/Cre		1.7 g/gCr	Na	141	mmol/L	Anti-SS-A Ab	>240	U/mL
Complete blood count			K	4.3	mmol/L	Anti-SS-B Ab	81.8	U/mL
WBC	3,100	/*μ*L	Cl	109	mmol/L	Anti-CCP-Ab	<0.6	U/mL
Lymphocyte	1,080	/*μ*L	LDH	199	U/L	Rheumatoid factor	<3	IU/mL
RBC	2.89 × 10^6^	/*μ*L	Nt-proBNP	460	pg/mL	MPO-ANCA Ab	1290	U/mL
Hemoglobin	8.5	g/dL	KL-6	259.6	U/mL	PR3-ANCA Ab	1.1	U/mL
Hematocrit	25.2	%	Haptoglobin	110	ng/mL	Anti-GBM Ab	<2.0	U/mL
MCV	87.2	fl	Erythropoietin	5.3	IU/mL	Cryoglobulin	(—)	
Platelet	186 × 10^3^	/*μ*L	TSH	7.11	*μ*IU/mL	Anti-CL.*β*2 GPI Ab	<1.2	U/mL
Reticulocyte	3.5	‰	FT3	3.1	pg/mL	C3	46.2	mg/dL
ESR	59	mm/hr	FT4	1.2	ng/dL	C4	22.5	mg/dL
CRP	0.35	mg/dL	Anti-TPO Ab	29	IU/mL	CH50	27	U/mL
			Anti-thyroglobulin Ab	222	IU/mL	C1q-IC	2.2	*μ*g/mL

## Data Availability

The data used to support the findings of this study are available from the corresponding author upon request.
